# Resting and Exercise Lactate Dynamics in Heart Failure: Guiding Therapeutic Optimization

**DOI:** 10.3390/biomedicines14040884

**Published:** 2026-04-13

**Authors:** Aurora Ferro, Andrea Segreti, Nardi Tetaj, Martina Ciancio, Simone Pasquale Crispino, Riccardo Cricco, Chiara Fossati, Fabio Pigozzi, Francesco Grigioni

**Affiliations:** 1 Cardiology Unit, Fondazione Policlinico Universitario Campus Bio-Medico, Via Alvaro del Portillo, 200, 00128 Rome, Italy; aurora.ferro@unicampus.it (A.F.);; 2 Research Unit of Cardiovascular Science, Department of Medicine and Surgery, Università Campus Bio-Medico di Roma, Via Alvaro del Portillo, 21, 00128 Rome, Italy; 3 Department of Movement, Human and Health Sciences, University of Rome “Foro Italico”, Piazza Lauro de Bosis, 15, 00135 Rome, Italy

**Keywords:** anaerobic threshold, cardiopulmonary exercise testing, heart failure, lactate, metabolic dysfunction, personalized therapy, risk stratification, ventilatory inefficiency

## Abstract

In heart failure (HF), elevated blood lactate levels, particularly during exercise or in advanced disease stages, reflect impaired tissue perfusion and altered metabolic regulation. Beyond its traditional role as a marker of anaerobic metabolism, lactate has emerged as a dynamic indicator of metabolic reserve and ventilatory control. This narrative review summarizes current evidence on lactate dynamics at rest and during exercise, highlighting their pathophysiological and clinical relevance. In HF patients, exercise-induced lactate accumulation occurs earlier and at lower workloads, reflecting impaired oxidative capacity and reduced peripheral oxygen utilization. This phenomenon is closely associated with ventilatory inefficiency, as demonstrated by the relationship between lactate levels and the VE/VCO_2_ slope during cardiopulmonary exercise testing (CPET). Emerging data suggest that lactate is not only a marker of disease severity but also a potential mediator of chemoreflex activation and abnormal ventilatory responses. Furthermore, both pharmacologic and non-pharmacologic interventions may influence lactate production and utilization, supporting its role as a potential tool for therapeutic monitoring. Overall, the integration of lactate assessment, particularly during exercise, into clinical evaluation may provide additional insight into disease mechanisms, improve risk stratification, and contribute to personalized therapeutic optimization in patients with HF.

## 1. Introduction

The concept of heart failure (HF) has evolved from being regarded exclusively as a hemodynamic syndrome to being recognized as a systemic metabolic disorder. This paradigm shift is attributable to mounting evidence showing that impaired energy utilization contributes to exercise intolerance, ventilatory inefficiency, and disease progression [1].

Conventional prognostic biomarkers, including natriuretic peptides and peak oxygen consumption, offer significant insights; however, they do not fully capture the dynamic metabolic adaptations that occur during periods of physical exertion. Among metabolic intermediates, lactate is distinctive in its role as a nexus between cellular redox state, peripheral oxygen delivery, and ventilatory control. Lactate was once regarded solely as an anaerobic byproduct; however, it is now understood to be a key metabolic substrate, signaling molecule, and potential driver of impaired ventilatory responses in HF [2,3].

Physiologically, ventilation is regulated by chemoreceptor sensing of arterial oxygen and carbon dioxide tension. Nevertheless, this framework is inadequate to explain the exaggerated ventilatory response observed in HF, wherein patients frequently exhibit an elevated VE/VCO_2_ slope and hyperventilation despite normal PaO_2_ and the absence of hypercapnia [4,5]. Experimental evidence demonstrates that rising lactate concentrations can directly stimulate carotid body activity, even in the absence of hypoxia, via lactate-sensitive olfactory receptors expressed on chemoreceptor cells [2]. Clinical observations further support this concept: arterial lactate increases during exercise correlate strongly with ventilatory inefficiency in HF patients, whereas peak lactate levels do not, suggesting that dynamic lactate kinetics rather than absolute lactate values are more closely related to abnormal ventilatory control [6].

This integrated physiometabolic perspective, encompassing cardiovascular, respiratory, and metabolic responses to exercise, provides a plausible mechanistic bridge between impaired cardiac output, early anaerobic metabolism, and the heightened chemoreflex drive that characterizes HF [7,8].

Supporting evidence shows that interventions capable of reducing lactate production, such as structured exercise training or metabolic modulators, can attenuate ventilatory overactivity and improve symptoms [9,10].

Taken together, these findings suggest that lactate may represent more than a terminal marker of hypoperfusion. Instead, it may serve as a dynamic biomarker of metabolic reserve and ventilatory stress, with potential implications for risk stratification and therapeutic tailoring. The present review examines the pathophysiological determinants of lactate accumulation in HF, its prognostic significance both at rest and during exercise, and the emerging role of pharmacologic and metabolic interventions in modulating lactate dynamics. The review proposes a framework for integrating lactate assessment into personalized HF management. This narrative review is based on a selective analysis of the literature, including key mechanistic, clinical, and translational studies, with particular emphasis on recent evidence and relevance to clinical practice.

## 2. Pathophysiology of Lactate Accumulation in Heart Failure

Lactate has traditionally been regarded as a product of anaerobic glycolysis. However, recent research has led to a re-evaluation of this metabolite, with it now being recognized as a versatile molecule that plays a critical role in cellular energy homeostasis, redox balance, and signaling [4,5].

Lactate has been proposed as a potential contributor to ventilatory inefficiency in HF; however, current evidence is predominantly associative, and direct mechanistic confirmation remains limited. Other established contributors, including ergoreflex activation, pulmonary vascular abnormalities, and altered ventilatory control, likely play a major role in this context [5].

In healthy conditions, cardiomyocytes primarily rely on fatty oxidation (50–90%) for energy, with pyruvate, originating from glycolysis and/or lactate conversion, contributing an additional 20–40%. In such conditions, the production and clearance of lactate are subject to stringent regulation by a concerted effort among the heart, liver, kidneys, and skeletal muscle, which function as pivotal organs in its metabolism [10].

Furthermore, lactate levels are influenced by hepatic function, as the liver plays a central role in lactate clearance through gluconeogenesis. In the context of HF, particularly in advanced stages, hepatic congestion and reduced perfusion may impair lactate clearance, contributing to elevated circulating levels independent of lactate production. This aspect is clinically relevant, as increased lactate levels in these settings may reflect both enhanced production and reduced clearance, thereby complicating their interpretation [11].

In right-sided HF, hepatic congestion may impair lactate clearance due to reduced hepatic function, whereas in left-sided HF, lactate elevation is more commonly driven by systemic hypoperfusion and increased anaerobic metabolism [12,13].

In stable chronic HF, lactate production is modestly increased (~1.5–2.5 mmol/L) because baseline oxygen delivery to tissues is usually preserved at rest. However, it has been demonstrated that lactate levels increase disproportionately during periods of exertion. This phenomenon can be attributed to chronic peripheral metabolic alterations, which in turn lead to reduced oxidative capacity [14].

Patients with HF develop a generalized muscle atrophy, characterized by an alteration in the distribution of muscle fibers, with a concomitant increase in the proportion of type II fibers (anaerobic, glycolytic) compared with type I fibers (aerobic, oxidative) [15].

**Figure 1:**
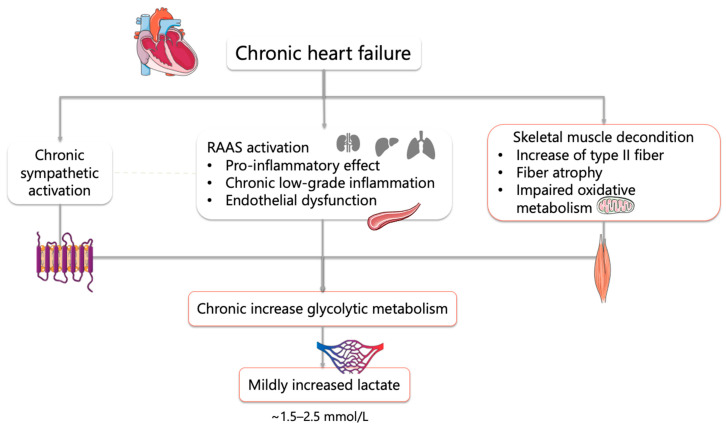
Physiopathology of increased lactate in chronic HF. Abbreviations: RAAS, renin–angiotensin–aldosterone system.

Figure 1 illustrates the complex interplay between central hemodynamic impairment and peripheral metabolic alterations that drive lactate accumulation in chronic HF.

Overall, lactate accumulation in chronic HF reflects the interplay between central hemodynamic impairment, leading to reduced cardiac output and oxygen delivery, and peripheral metabolic alterations, including skeletal muscle dysfunction, reduced oxidative capacity, and a shift toward glycolytic metabolism. These mechanisms collectively promote early activation of anaerobic pathways and impaired lactate clearance, ultimately contributing to abnormal lactate dynamics.

In acute HF, especially in cases of cardiogenic shock, lactate levels can escalate rapidly to 4–6 mmol/L, and levels exceeding 8–10 mmol/L are indicative of profound shock and are associated with a high mortality rate [16]. This marked increase reflects hypotension, hypoxia, and systemic hypoperfusion. Consequently, there is a critical reduction in oxygen delivery, resulting in a metabolic shift to anaerobic glycolysis and a substantial increase in lactate production [17].

It is important to note that, in cases of end-stage HF, elevated lactate levels are associated with a worsening INTERMACS profile [18].

## 3. Clinical and Prognostic Implications of Lactate Elevation

In HF, a multitude of pathophysiological derangements disrupt lactate homeostasis, resulting in premature and excessive lactate production, both at rest and during exercise, particularly in patients with advanced HF [13,14,15,19].

During exercise, patients with HF exhibit earlier lactate accumulation due to impaired oxygen delivery and reduced metabolic reserve, which contributes to exercise intolerance and symptom burden [20,21]. The clinical manifestation is characterized by the presence of symptoms, including fatigue, dyspnea, and markedly reduced exercise tolerance, common to both HF with reduced ejection fraction (HFrEF) and HFpEF.

During periods of rest, lactate abnormalities in HF are generally less pronounced compared with those observed during exercise. However, this does not exclude their clinical relevance.

In a cohort of patients suffering from both acute and chronic HF, elevated levels of the glycolytic intermediate lactate were found to be independently associated with a range of markers of ventilatory inefficiency, pulmonary congestion, and metabolic stress. This association remained significant even in the absence of hypotension [22].

It is important to note that lactate may have neurohumoral implications beyond its metabolic role. Experimental and clinical evidence suggests that, in patients with HF, impaired cardiac output during exercise leads to increased levels of both lactate and hydrogen ions (H^+^). These elevated levels in turn stimulate carotid chemoreceptors, thereby heightening ventilatory drive and prompting a reduction in the arterial partial pressure of carbon dioxide (PaCO_2_) [6].

In acute HF, hyperlactatemia is present in approximately 30–50% of patients, and lactate is a dynamic indicator of therapeutic response, particularly in acute settings such as cardiogenic shock patients [23].

Clinical trials and meta-analyses demonstrated that lactate clearance, defined as the relative reduction in lactate over time, is a superior predictor of survival compared with a single lactate measurement. Patients exhibiting impaired lactate clearance consistently demonstrate significantly worse outcomes, even when initial lactate levels are not markedly elevated [24,25,26,27].

While the prognostic significance of elevated lactate levels is well established in acute HF, evidence in the context of chronic HF, particularly during stable phases, is comparatively limited and less systematically studied. Nevertheless, emerging data suggest that hyperlactatemia may carry meaningful prognostic and functional implications even in chronic settings [13,16]. In stable chronic HF, elevated exercise lactate levels are associated with reduced functional capacity and adverse clinical profiles [20]. In more acute-on-chronic scenarios, elevated admission lactate remains a strong predictor of early mortality, bridging the continuum between chronic and acute disease states [12,17].

## 4. Exercise Lactate and CPET in Heart Failure

### 4.1. Altered Lactate Physiology During Exercise

In healthy individuals, resting lactate levels are typically low and stable. Lactate accumulation during exercise follows a predictable, workload-dependent pattern, with a delayed inflection corresponding to the anaerobic threshold and efficient clearance during recovery [28,29].

Conversely, patients with HF exhibit significantly altered lactate kinetics. Although resting lactate concentrations may remain within the normal range in clinically stable HF, exercise unmasks a reduced metabolic reserve. Lactate accumulation occurs earlier and at lower workloads, reflecting reduced metabolic reserve [12,28,30]. This finding is indicative of impaired oxygen delivery, mitochondrial dysfunction of the skeletal muscle, altered fiber-type composition, and a consequent reliance on anaerobic glycolysis [18].

The anaerobic (lactate) threshold (AT) is defined as the exercise intensity at which there is an exponential accumulation of blood lactate, marking the transition from predominantly aerobic metabolism to significant anaerobic metabolism [31]. In patients with HF, this threshold is attained at a reduced absolute work rate and at an earlier point during exertion compared with healthy individuals [32].

Exercise training is one therapy known to reverse these abnormalities, albeit partially. There is a considerable body of evidence indicating that structured training programs result in a delay in the onset of lactate accumulation during submaximal exercise in HF [12,33,34,35].

The net effect of these adaptations is that trained HF patients are able to exercise at higher intensities before lactate begins to accumulate, effectively raising their anaerobic threshold [12,36,37].

### 4.2. Lactate and Ventilatory Inefficiency

CPET represents the gold standard for functional and prognostic assessment in HF, providing an integrated evaluation of cardiovascular, respiratory, and peripheral responses to exercise [38]. It should be noted that CPET-derived parameters may vary according to exercise modality (cycle ergometer vs. treadmill), which can influence peak VO_2_ values and ventilatory responses [39].

Recent studies have further refined the prognostic and pathophysiological role of CPET in HF, supporting its integration into routine clinical assessment [40,41].

Peak VO_2_ and AT are well-established prognostic indicators that are used to evaluate disease severity and even to guide transplant candidacy. A depressed peak VO_2_ (e.g., <~15 mL/kg/min, or <~10 in severe HF) coupled with an early AT defines a high-risk patient. In addition, an abnormally steep ventilatory equivalent for carbon dioxide slope (VE/VCO_2_), for example, a slope greater than 34, is indicative of ventilatory inefficiency and is associated with elevated filling pressures and adverse outcomes [42,43]. Although VE/VCO_2_ is not measured from lactate, its elevation frequently parallels metabolic stress and impaired perfusion [31,32,44,45]. However, VE/VCO_2_ values may vary across different HF phenotypes and patient populations and should be interpreted within the appropriate clinical context [46].

Furthermore, CPET interpretation relies predominantly on gas-exchange-derived variables, whose reliability may be compromised in advanced HF by ventilatory instability and abnormal breathing patterns, particularly exercise oscillatory ventilation (EOV) or periodic breathing [28,38,47]. In this context, blood lactate assessment offers a complementary and, in selected conditions, indispensable metabolic perspective [48].

The concept of a dual anaerobic threshold further integrates lactate into CPET interpretation. The first threshold corresponds to early metabolic lactate accumulation, reflecting limited peripheral oxidative capacity and early glycolytic activation. The second threshold, traditionally associated with RCP, reflects overt metabolic acidosis and buffering exhaustion. Evidence indicates that HF patients in whom both thresholds can be identified exhibit better survival, whereas the absence of one or both thresholds portends a worse prognosis [48]. In patients with EOV or advanced disease, the initial lactate-defined threshold frequently predominates. In contrast, the secondary threshold may be diminished or absent, a phenomenon that can result from premature termination of the test [44].

A contemporary, multiparametric perspective on exercise intolerance in HF is best aligned with an integrated interpretation of CPET incorporating lactate kinetics alongside ventilatory and hemodynamic variables [38,49].

### 4.3. Lactate Assessment During CPET

The assessment of blood lactate levels can be achieved through the implementation of diverse methodological approaches, which differ in terms of invasiveness, temporal resolution, and physiological representativeness. Conventional blood-based measurements are considered the reference standard in both clinical and research settings [50].

Arterial sampling is recognized as the most accurate method of estimating systemic lactate concentration; however, it is an invasive procedure and is not well suited to repeated measurements during exercise. Venous lactate sampling is a less invasive procedure, but it is subject to variation due to regional lactate extraction and delayed equilibration, particularly during dynamic exercise conditions. Capillary sampling from the fingertip or earlobe has become the preferred method during CPET, owing to the following reasons: its feasibility and minimal invasiveness, and the fact that it exhibits good agreement with arterial measurements when standardized protocols are applied [51].

Conventional blood-based measurements are being complemented by emerging technologies that are exploring alternative approaches for lactate monitoring. The use of microneedle-based biosensors and transdermal detection systems for continuous lactate sensing has demonstrated promising concordance with blood lactate dynamics during exercise, thereby offering the potential for minimally invasive, real-time monitoring. In a similar vein, research is underway into the potential of optical and enzymatic wearable sensors to detect lactate in interstitial fluid or sweat. However, it is important to note that the clinical applicability of these sensors in HF populations remains to be validated [52,53].

The clinical interpretation of lactate levels is influenced by several methodological factors. Small but consistent differences between sampling sites (e.g., earlobe vs. fingertip) have been reported, particularly during dynamic exercise conditions.

Differences in sampling site (arterial, venous, or capillary), timing of collection, and analytical techniques may significantly affect measured values [45,46].

In addition, pre-analytical conditions, such as delayed sample processing, can lead to falsely elevated levels due to ongoing glycolysis. These aspects underscore the need for standardized protocols and careful interpretation of lactate measurements in clinical practice [47,48].

### 4.4. Phenotypic Differences (HFrEF vs. HFpEF)

HF with reduced ejection fraction (HFrEF) provides an illustration of the pattern of early anaerobic metabolism described above, driven by a markedly reduced cardiac output reserve. In patients with HFrEF, reduced muscle perfusion and reliance on glycolysis result in the production of lactate following the commencement of exercise [54]. Peripheral skeletal muscle changes, such as a shift toward fast-twitch glycolytic fibers and reduced oxidative enzyme activity, further predispose to early lactate buildup [55]. Consequently, patients with HFrEF frequently reach their lactate threshold rapidly and exhibit a constrained peak VO_2_ [56,57]. However, in advanced HF, there may be such a significant compromise to muscle perfusion that lactic acidosis may be absent at rest despite very low cardiac output. A subset of patients in cardiogenic shock can have normal lactate levels until very late stages [12,58]. This finding (Adamo et al. 2017) emphasizes that lactate elevation in chronic HFrEF tends to manifest during exertion rather than at rest, and its appearance at rest is a late marker of circulatory decompensation [13].

The clinical presentation of HF with preserved ejection fraction (HFpEF) is accompanied by a distinctive lactate profile. Despite a normal left ventricular ejection fraction, patients with HFpEF exhibit significant exercise intolerance and metabolic inefficiency. Furthermore, the subjects demonstrated a reduced cardiac output during the exercise period. This is driven by a combination of impaired cardiac output reserve, chronotropic incompetence, and reduced peripheral oxygen extraction [59], leading to abnormal oxygen extraction by muscles. It is noteworthy that patients with HFpEF in the 2024 study exhibited elevated mixed-venous O_2_ saturation levels and a reduced oxygen extraction ratio during exercise, suggesting that their muscles were not optimally utilizing the oxygen supplied. This finding suggests the presence of peripheral metabolic inflexibility, a condition potentially resulting from reduced muscle mitochondrial function or microvascular dysfunction, in addition to the central limitation imposed by a stiff heart. Collectively, these central and peripheral limitations result in an early switch to anaerobic metabolism. HFpEF patients may accumulate lactate at low exercise intensities (even during daily activities) that would be purely aerobic for healthy individuals. The phenomenon of “hyperlactatemia” in HFpEF demonstrates a robust correlation with diminished peak VO_2_ and exercise capacity. Indeed, elevated exercise lactate levels in HFpEF are now widely recognized as a marker of hemodynamic and cardiometabolic derangement.

HFpEF frequently exhibits an exaggerated VE/VCO_2_ slope (ventilatory inefficiency) that is comparable to that seen in HFrEF, partly due to the presence of frequent pulmonary hypertension and obesity-related deconditioning in this population. Consequently, a preserved ejection fraction does not prevent patients from experiencing limitations driven by lactate. HFpEF emphasizes the crucial role of peripheral metabolism and chronotropic response in exercise tolerance. These findings imply that therapeutic interventions aimed at enhancing peripheral fitness and metabolic flexibility (e.g., endurance training, muscle strengthening, or metabolic modulators) are as crucial as addressing cardiac output in the management of HFpEF.

## 5. Pharmacologic Modulation of Lactate Metabolism in Heart Failure

The following sections aim to investigate how guideline-directed medical therapies (GDMT) interact with lactate metabolism, both influencing and being influenced by its dynamics.

The available evidence on pharmacologic modulation of lactate metabolism in HF is heterogeneous, including both studies directly measuring lactate levels and others providing indirect evidence through CPET-derived parameters or experimental models. This distinction is important when interpreting the clinical relevance of these findings.

### 5.1. ACE Inhibitors and ARBs

ACEis have been shown to improve myocardial energy metabolism, shifting the failing heart from net lactate production to net lactate uptake. In patients with chronic HF, the acute administration of the ACEi Cilazapril resulted in an increased myocardial lactate extraction both during periods of rest and during exercise. In several patients, this shift in myocardial lactate extraction towards net uptake indicated a shift towards more efficient aerobic metabolism [60].

The administration of enalapril over a prolonged period has been demonstrated to induce favorable alterations in the distribution of lactate dehydrogenase (LDH) isoenzymes. In patients with HF, enalapril administration resulted in a substantial increase in the proportion of LDH1 and a concomitant reduction in LDH5, indicative of a transition toward aerobic metabolism [61].

ARBs exert analogous effects. In rat models of HF, chronic Losartan therapy has been shown to reduce lactate accumulation in coronary effluent and to decrease myocardial tissue lactate content by approximately 57%, while simultaneously increasing ATP (~33%) and glycogen (~43%), reflecting improved high-energy phosphate metabolism [62].

Despite the rarity of direct blood lactate measurement in contemporary HF trials, mechanistic and translational data support the hypothesis that RAAS blockade enhances myocardial lactate utilization and delays exercise-induced hyperlactatemia, contributing to improved exercise tolerance and clinical outcomes [63,64].

### 5.2. Angiotensin Receptor–Neprilysin Inhibitors (ARNIs)

In patients with HFrEF, sacubitril/valsartan has been associated with improvements in functional capacity, as evidenced by significant increases in peak VO_2_, ventilatory efficiency, and VO_2_ at the anaerobic threshold. Prospective and observational studies have shown 15–17% gains in peak VO_2_ and significant reductions in VE/VCO_2_ slope [65,66].

Despite the absence of direct blood lactate measurements, the consistent upward shift in anaerobic threshold VO_2_ provides indirect evidence of reduced lactate production during exercise. Mechanistically, ARNI therapy enhances natriuretic peptide signaling, promoting mitochondrial biogenesis and increasing oxidative capacity in skeletal muscle, thereby facilitating aerobic substrate utilization [67].

This hypothesis is supported by preclinical models demonstrating that sacubitril/valsartan enhances exercise endurance and reduces the respiratory quotient, indicating a shift towards greater reliance on oxidative metabolism and a decrease in glycolytic activity [68].

The current clinical and translational evidence indicates that sacubitril/valsartan enhances oxidative metabolism and likely contributes to delayed exercise-induced hyperlactatemia in HFrEF, even in the absence of direct lactate measurements.

### 5.3. SGLT2 Inhibitors

SGLT2i has been shown to induce a metabolic shift that is analogous to the fasting state, characterized by enhanced rates of fatty acid and ketone oxidation and reduced reliance on glycolysis, a profile that is expected to result in a reduction in lactate production.

In the CAMEO-DAPA trial, patients with HFpEF treated with dapagliflozin exhibited significantly lower arterial lactate concentrations during submaximal exercise, despite no change in peak VO_2_. This finding indicates improved oxidative substrate utilization and a delayed transition to anaerobic metabolism [69].

Complementary data from HFrEF trials, such as EMPA-TROPISM, demonstrate that empagliflozin increases peak VO_2_ and exercise time at the ventilatory threshold. This indirect evidence suggests that lactate accumulation under exertion is attenuated [70].

A recent meta-analysis confirmed these findings, reporting consistent improvements in anaerobic threshold across studies of SGLT2i in HF [71].

While at rest, changes in circulating lactate appear negligible; both translational and preclinical lines of evidence suggest that SGLT2i reduce glycolytic flux and promote oxidative metabolism, thereby limiting lactate generation [72]. Mechanistic studies in diabetic and failing hearts demonstrate reduced myocardial lactate production and enhanced fatty acid oxidation following exposure to empagliflozin. This process is partly mediated by Na^+^/H^+^ exchanger (NHE-1) inhibition [73].

### 5.4. Mineralocorticoid Receptor Antagonists (MRAs)

MRAs inhibit the effects of aldosterone, thereby reducing myocardial fibrosis and improving endothelial function [74].

It is possible that these changes could theoretically enhance skeletal muscle perfusion and oxygen utilization during exercise. Nevertheless, there is an absence of direct evidence to suggest that MRAs modulate lactate levels in either a resting or exercise state. In the Aldo-DHF trial (HFpEF patients), 12 months of spironolactone treatment resulted in improvements in diastolic function. However, no significant changes were observed in maximal exercise capacity (peak VO_2_), indicating that the effects on exercise-related lactate production were minimal [75].

### 5.5. Beta-Blockers

By blocking β2-adrenergic signals, β-blockers have been shown to reduce sympathetic stimulation of skeletal muscle glycolysis, thereby decreasing exercise-induced lactate output [76]. In failing hearts, β-blockade has been demonstrated to reduce myocardial oxygen demand and shift net metabolism towards increased lactate uptake. For instance, acute metoprolol administration in patients with congestive HF has been shown to promote net myocardial lactate consumption over production [77].

In accordance with these effects, chronic β-blockade in HF has been observed to preserve (or even improve) aerobic capacity, as evidenced by pooled trials demonstrating no decline in peak VO_2_ (and an improvement in NYHA functional class) on β-blockers [78].

In the context of a CPET, the ventilatory (anaerobic) threshold remains largely unaltered. Concurrently, the peak O_2_ pulse (VO_2_/HR) frequently exhibits an upward trend, attributable to an augmentation in stroke volume that occurs alongside a decrease in heart rate [79].

In patients with HFpEF, the administration of β-blockers has been shown to reduce the increase in VO_2_ and O_2_-pulse levels in a specific study. Conversely, an alternative analysis has demonstrated no alteration in peak VO_2_ levels, but rather an elevated anaerobic threshold in response to β-blockade [80,81].

It is important to note that β1-selective blockers spare β2-mediated vasodilation during exercise (and carvedilol’s α1-blockade further counteracts sympathetic vasoconstriction), thus ensuring that muscle perfusion is not compromised [79].

It is also noteworthy that these effects do not appear to compromise the process of training adaptation. HF patients on β-blockers still achieve normal improvements in lactate threshold and peak VO_2_ with rehabilitation, indicating preserved metabolic flexibility.

Taken together, although direct evidence on lactate modulation by β-blockers in heart failure remains limited, the available clinical and mechanistic data consistently suggest that chronic β-blockade reduces adrenergic lactate surges, enhances myocardial lactate utilization, and preserves exercise-induced metabolic adaptations, thereby supporting improved functional capacity and prognosis.

### 5.6. Iron Supplementation

The identification of lactate as both a marker and a mediator of metabolic inefficiency in HF also provides a framework for the interpretation of the effects of iron repletion. In iron-deficient HFrEF, impaired mitochondrial function forces skeletal muscle to rely on glycolysis, thereby enhancing lactate generation during exertion [82,83]. In a randomized controlled trial, intravenous ferric carboxymaltose significantly reduced exercise-induced lactate production in comparison with a placebo, despite no alterations in hemoglobin or systemic inflammation. This finding suggests that the therapeutic benefit of iron extends beyond hematopoiesis, directly restoring oxidative metabolism and limiting anaerobic lactate accumulation [84]. Consequently, lactate profiling has the potential to serve as a sensitive endpoint, capable of reflecting disease severity and providing a means to monitor the metabolic impact of targeted interventions, such as iron supplementation.

### 5.7. Measurement Strategy: Resting vs. Exercise Lactate

In cases of chronic HF, lactate levels are frequently within normal limits during periods of rest. However, the use of exercise lactate profiling during CPET has been shown to be a sensitive indicator of metabolic inefficiency and the response to therapeutic intervention [12,29,85].

The incorporation of lactate endpoints into HF trials has the potential to elucidate therapy-specific metabolic effects and thus guide personalized management.

The following Table 1 summarizes the clinical and preclinical evidence on the modulation of lactate by pharmacological therapy in HF. Direct evidence is defined as studies that explicitly measure lactate levels (e.g., myocardial or arterial lactate levels, balance, or clearance). Indirect evidence is defined as data derived from markers associated with lactate metabolism, including ventilatory efficiency, anaerobic threshold, and LDH isoenzyme distribution.

## 6. Future Directions and Perspectives

In summary, lactate in HF represents a dynamic integrative biomarker that reflects the complex interplay between hemodynamic impairment, metabolic dysfunction, and altered ventilatory control.

Future research should move beyond the current paradigm of considering lactate as a generic stress marker to achieve a more nuanced understanding of its role in physiological processes. Instead, the focus should be on testing it as a dynamic biomarker embedded in precision HF care.

Conducting prospective studies to standardize lactate sampling at rest and during CPET is essential. Moreover, it is important that these studies define clinically meaningful thresholds and trajectories (e.g., early hyperlactatemia vs. blunted peak lactate) [12].

Finally, it is pivotal that these indices be integrated with established risk markers, such as natriuretic peptides, peak VO_2_, VE/VCO_2_ slope, and imaging. Concurrently, mechanistic and interventional trials should evaluate whether therapies that modulate metabolism and chemoreflex activity, including SGLT2 inhibitors, ARNI, RAAS blockade, iron repletion, and β-blockers, can be better titrated or selected using lactate-based phenotyping [5,6,38].

A particularly attractive avenue for future research is the identification of HF subgroups with lactate-driven chemoreceptor sensitization and ventilatory inefficiency, who might benefit from targeted approaches such as structured aerobic training, high-intensity interval rehabilitation, carotid body modulation, or novel metabolic agents. Incorporating point-of-care and serial lactate measurements into HF programs, including cardiac rehabilitation, may facilitate real-time monitoring of metabolic reserve and therapeutic response. This may, in turn, allow the development of patient-specific treatment algorithms guided by lactate measurements [38,85].

HF is increasingly recognized as a systemic metabolic disorder, in which impaired energy utilization and abnormal peripheral responses contribute to symptoms, ventilatory inefficiency, and disease progression. Within this framework, lactate emerges as a dynamic integrative biomarker linking hemodynamic impairment, skeletal muscle dysfunction, mitochondrial alterations, and chemoreflex activation.

Evidence from both acute and chronic HF indicates that lactate elevation, at rest, during exercise, or due to impaired clearance, reflects not only tissue hypoperfusion but also broader metabolic and neurohumoral dysregulation. Exercise-induced lactate dynamics serve as a sensitive marker of reduced metabolic reserve and early anaerobic activation and are closely associated with ventilatory inefficiency, reduced functional capacity, and adverse outcomes across HF phenotypes.

CPET provides a unique opportunity to integrate lactate kinetics within cardiopulmonary responses, allowing a more comprehensive evaluation of exercise intolerance beyond conventional gas-exchange variables. Emerging evidence further suggests that contemporary pharmacologic and non-pharmacologic therapies may modulate lactate production and utilization, supporting its role not only as a marker of disease severity but also as a potential indicator of therapeutic response.

From a clinical standpoint, lactate assessment may represent a complementary tool in the evaluation of patients with HF. During CPET, serial lactate measurements may provide additional information on metabolic thresholds and peripheral adaptation, potentially refining the interpretation of exercise limitation beyond ventilatory parameters alone. In acute decompensated HF, elevated lactate levels may reflect both impaired tissue perfusion and reduced clearance, thereby offering further insight into the severity of hemodynamic compromise. In the setting of cardiac rehabilitation, lactate profiling may also help monitor metabolic response to training over time. Importantly, lactate assessment should be considered complementary to established clinical and CPET-derived markers rather than used in isolation.

Despite these advances, lactate assessment remains underutilized in routine HF care, largely due to the lack of standardized protocols, validated thresholds, and prospective data. Future research should aim to define clinically meaningful lactate profiles at rest and during exercise, and to determine whether lactate-guided strategies can improve risk stratification and therapeutic optimization.

Overall, these findings support a paradigm shift in which lactate is recognized as a dynamic biomarker of metabolic reserve and ventilatory stress in HF. Its integration into clinical practice, particularly in the context of exercise testing, may help bridge the gap between pathophysiological insight and personalized management.

This review has several limitations. As a narrative review, it is subject to potential selection bias. In addition, the available evidence on lactate in HF is heterogeneous, with limited prospective and standardized data, particularly in the context of exercise testing. These limitations should be considered when interpreting the findings and their potential clinical implications.

## Figures and Tables

**Table 1:** Summarizes the available evidence on lactate modulation in HF, distinguishing between observational studies, randomized controlled trials, and preclinical data.

Drug/Class	Population	Study Design	Type of Evidence	Result on Lactate	Level of Evidence
ACEi (Cilazapril) [60]	Patients with CHF	Observational study	Direct (myocardial lactate measurement)	↑ myocardial lactate extraction (shift from net lactate production to net uptake), indicating improved myocardial oxidative metabolism	Moderate
ACEi (Enalapril) [61]	Patients with CHF	Observational study	Indirect (LDH isoenzymes related to lactate metabolism)	↑ LDH1 and ↓ LDH5 → indicative of reduced lactate production	Moderate
ARB (Losartan)	Murine models of HF	Preclinical study	Direct (lactate measurement)	↓ lactate in coronary effluent and myocardial tissue (−57%) + ↑ ATP and glycogen	Low
ARNI (Sacubitril/Valsartan) [62,68]	Patients with HFrEF	Observational study	Indirect (anaerobic threshold, ventilatory efficiency)	↑ peak VO_2_ and anaerobic threshold, ↓ VE/VCO_2_ slope → suggested reduced lactate accumulation	Moderate
SGLT2i (Dapagliflozin) [69]	Patients with HFpEF	CAMEO-DAPA trial	Direct (arterial lactate measurement)	↓ arterial lactate concentrations during submaximal exercise	High
SGLT2i (Empagliflozin) [70]	Preclinical models (CHF and diabetes)	Preclinical/mechanistic study	Direct (myocardial lactate measurement)	↓ myocardial lactate production, ↑ fatty acid, and ketone oxidation	Low
MRAs (Spironolactone) [75]	Patients with HFpEF (Aldo-DHF trial)	Randomized controlled trial	Indirect (exercise capacity and diastolic function)	No significant change in peak VO_2_ → no evidence of lactate modulation	High
β-blockers (Metoprolol) [77]	Patients with congestive HF	Observational study	Direct (myocardial lactate balance)	Shift from net lactate production to net lactate consumption	Moderate
Iron supplementation (Ferric carboxymaltose) [82]	Patients with HFrEF and iron deficiency	Randomized controlled trial	Direct (exercise-induced lactate measurement)	↓ Exercise-induced lactate production, independent of Hb or inflammation	High

## Data Availability

No new data were created or analyzed in this study. Data sharing is not applicable.
